# Preventive oral health practices of school pupils in Southern Nigeria

**DOI:** 10.1186/1472-6831-14-83

**Published:** 2014-07-07

**Authors:** Morenike O Folayan, Mohammad R Khami, Nneka Onyejaka, Bamidele O Popoola, Yewande Isabella Adeyemo

**Affiliations:** 1 Department of Child Dental Health, Obafemi Awolowo University, Ile-Ife, Nigeria; 2 Research Center for Caries Prevention, Community Oral Health Department, School of Dentistry, Tehran University of Medical Sciences, Tehran, Iran; 3 Department of Child Dental Health, University of Nigeria Teaching Hospital, Enugu, Nigeria; 4 Department of Child Oral Health, University of Ibadan, Ibadan, Nigeria; 5 Department of Child Oral Health, University College Hospital, Ibadan, Nigeria

## Abstract

**Background:**

One of the goals of the World Health Organisation goal is to ensure increased uptake of preventive oral self-care by 2020. This would require the design public health programmes that will ensure children place premium on preventive oral health care uptake. One effort in that direction is the need for countries to define baseline measures on use of preventive oral self-care measures by their population as well as identify factors that impact on its use. This study aims to determine the prevalence and the impact of age and sex on the use of recommended oral self-care measures by pupils in Southern Nigeria.

**Methods:**

Pupils age 8 to 16 years (N = 2,676) in two urban sites in Southern Nigeria completed a questionnaire about recommended oral self-care (use of fluoridated toothpaste, flossing, regularity of consuming sugary snacks between main meals), time of the last dental check-up and cigarette smoking habit. Chi square was used to test association between age (8-10years, 11–16 years), sex, and use of recommended oral self-care. Logistic regression analysis was used to determine the predictors of use of recommended oral self-care.

**Results:**

Only 7.8% of the study population practiced the recommended oral self-care. Older adolescents had an 8.0% increased odds (OR: 1.08; CI:0.81–1.43; p = 0.61) and males had a 20.0% decreased odds (OR: 0.80; CI:0.60-1.06; p = 0.12) of practicing recommended oral self-care though observed differences were not statistically significant. Very few respondents (12.7%) had visited the dental clinic for a check-up in the last one year. Majority of the respondents (92.2%) were non-smokers.

**Conclusions:**

The use of a combination of oral self-care approaches was very low for this study population. Age and sex were predictive factors for the use of components of the oral self-care measures but not significant predictors of use of recommended oral self-care. Future studies would be required to understand ‘why’ and ‘how’ age and sex impacts on the use of caries preventive oral self-care measures to be able to design effective prevention educational programmes for the study population.

## Background

The aetiology of most oral diseases are behaviour-related [1-3]. These can be prevented by adopting and maintaining healthy habits, ensuring oral self-care [3-5] and accessing regular dental check-ups [6,7]. These same principles are essential for caries control, a disease that is highly prevalent in children in many parts of the world. Thus where compliance with oral hygiene instruction and healthy habits is poor, instituted caries control measures may fail independent of the effectiveness of the preventive method used [8].

Oral health habits of significant importance in caries prevention in children include the use of fluoridated toothpastes [9-12], twice daily brushing [13,14], the use of dental floss for interdental cleaning, restriction in consumption of refined carbohydrate in-between meals [15-17] and regular dental checkups. In addition, there is the need to pay close attention to prevention of tobacco smoking due to its detrimental effects on general and oral health [18,19], and the incidence of oral cancers [20,21].

In Nigeria, though the prevalence of dental caries is low, the rate of unmanaged dental caries is high resulting in significantly high risk for odontogenic infection [22,23]. Reasons for poor utilization of oral health care services has more to do with an unperceived need for these services due to the absence of pain. The risk of developing a spiral effect that results in significant increase in caries prevalence and severity due to untreated dental caries is high: children with untreated dental caries have a five-fold increased risk of having a new lesion over a three year period [24]. These same children may one day become parents who could infect their children with cariogenic organisms. While this hypothesis remains untested, the feasibility of this makes it an imperative for public health workers to place high premium on the institution of preventive oral health care in children in line with the WHO goal of ensuring increased uptake of preventive oral self-care by 2020 [25]. It is therefore important to strategically design public health programmes that will ensure children place premium on preventive oral health care uptake.

A first effort in this direction will be to understand the current preventive oral health care practices of children, the level of influence of care givers and significant others, and impact of proxy to dental care services in promoting oral self-care of children. This study shall specifically focus on determining the prevalence of use of recommended oral self-care measures – a combination of prescribed dental caries prevention measures - by primary and secondary school pupils in urban centres in Southern Nigeria where dental care services is available; and the possible impact of age and sex on the likelihood of using these oral self-care measures.

## Methods

### Background information

This study was conducted in Nigeria, a highly populous country with young persons and adolescents constituting 55% and 23% of its population respectively. Thirty million children are in school [26] representing only 62.1% and 44.1% of eligible primary and secondary school students [27]. The country is made up of 36 states divided into six geopolitical zones. Three of geopolitical zones are in either of the two regions (Northern and Southern Nigeria). The culture, norms, dietary habits, beliefs and practices of the two regions differ significantly, though there is more homogeneity within the regions. The number of decayed, missing and filled teeth (DMFT) are higher in children from Northern Nigeria [28].

### Study design

This is a cross sectional study enrolling a sample of pupils in primary and secondary schools in two states in Southern Nigeria: Ibadan and Enugu. The two states were selected from the 18 states in Southern Nigeria based on ease of access by the study team. The two states represented the two major cultural groups in southern Nigeria – Yorubas and Igbos.

In each state, pupils from primary and secondary schools in the three local government areas that make up Enugu metropolis and the five local government areas that make up Ibadan metropolis were randomly selected using a multi-prong approach.

The sample was first proportionally distributed amongst the Local Government Areas and then, proportionately distributed between the private and public schools in the Local Government Areas. Each public and private secondary school from which participants emerged were selected from the sampling frame based on a constant of K = 10 (The constant K was chosen to be 10 based on the least number of schools in a local government such that every 10th school in each local government was selected for the study).

In each school, the classes with the highest number of pupils who met the age eligibility criteria were selected for the study. Children who fall within the age range for the study were recruited into the study. Recruitment in each school continued till the sample size for the school was reached.

### Sample size

The minimum sample size calculated for the study was 1,333 for Enugu and 1,228 for Ibadan. The sample size was calculated using a standard normal deviate at 95% confidence interval, standard normal deviate of 1.96 when the beta error allowed is 10% and the power of the study is 90%. The prevalence of caries of 15.0% in Enugu [29] and 10.8% in Ibadan [30] was used for the sample size estimation.

### Target population

The study population consisted of children aged 8–16 years recruited from 34 primary schools and three secondary schools in Enugu metropolis, and 21 secondary schools and three primary schools in Ibadan metropolis. The minimum age for study participation was fixed at age 8 years so as to ensure appropriate responses for each of the items in the study questionnaire could be generated. Informed consent for study participation was sought from the parents of all the children. Assent was also sought from children age 12 and above. Only children who were willing to participate in the study were recruited into the study. Pupils with special needs (physically, medically and mentally challenged) were excluded for the study.

### Questionnaire administration

The study adopted the questionnaire utilized by Khami et al. [31] for the assessment of preventive oral self-care in dental students. Questionnaires were self-administered and had fixed responses. A team of two researchers were trained to administer the questionnaire in each of the classrooms where students were recruited. The researchers served as guide to the pupils and answered any clarifying questions: they went through the questionnaire with the class and gave expounded explanations for each of the six questions and their options.

The questions asked for specific information on frequency of tooth brushing, use of dental floss, consumption of sugar-containing snacks or drinks between main meals, smoking of cigarette, and time of last dental check-up. Researchers provided specific guide on the use of fluoride containing toothpaste by highlighting the various types of fluoride and non-fluoride containing toothpastes in the market. Where alternatives to toothpastes were used for tooth cleansing, respondents were asked to tick the option that reflected they do not use fluoride containing toothpaste for tooth cleaning.

The purpose of the study and issues of confidentiality were highlighted on each questionnaire. Instructions on how to complete the questionnaires were also clearly stated. Pupils were asked to refrain from recording their names on the questionnaire for reasons of confidentiality. They were however asked to indicate their sex (male or female) and age (at last birthday in years). The questionnaire requested information on respondents’ oral health behaviour. The questionnaire assessed:

1. *Oral Self*-*Care*: Recommended oral self-care was defined as a composite score derived from indications of brushing teeth at more than once a day, use of fluoridated toothpaste, and consumption of sugary snacks between main meals less frequently than once a day [20,32,33]. Each respondent needs to have met the three criteria to be categorised as practicing recommended oral self-care

2. *Tooth*-*brushing*: Respondents were also asked to indicate the frequency of tooth brushing using the following alternatives – irregularly or never, Once a week, a few (2–3) times a week; once a day, and more than once a day. Respondents who chose the options ‘i*rregularly or never*, *Once a week*, *a few* (*2*–*3*) *times a week*; *once a day*’ were classified as not having undertaken preventive dental care.

3. *Use of fluoridated toothpaste*: Respondents were also asked to indicate the frequency of use of fluoridated toothpaste when tooth brushing using the following alternatives – *Always*, *quiet often*, *seldom*, *not at all*. Respondents who chose the options ‘*quiet often*, *seldom*, *not at all*’ were classified as not having undertaken preventive dental care.

4. *Consumption of sugary snacks between meals*: Respondents were also asked to indicate the frequency of consuming sugar-containing snacks or drinks between your main meals using the following alternatives – *About 3 times a day or more*, *about twice a day*, *about once a day*, *0ccasionally*; *not every day*, *rarely or never eat between meals*. Respondents who chose the options ‘*About 3 times a day or more*, *about twice a day*, *about once a day*’, were classified as not having undertaken preventive dental care.

5. *Use of dental floss*: Respondents were also asked to indicate how often dental floss was used for to clean the teeth using the following alternatives – *Not at all*, *occasionally*, *a few* (*2*–*3*) *times a week*, *once in a day*, *more than one time in a day*. Respondents, who chose the options ‘*Not at all*, *occasionally*, *a few* (*2*–*3*) *times a week*’, were classified as not having undertaken preventive dental care.

6. *Dental service utilization*: Respondents were also asked to indicate the time of the last check-up using the following alternatives - *within the last 6 months*, *more than 6 months to one year ago*, *more than 1 to 2 years ago*, *more than 2 to 5 years ago*, *more than 5 years*, *never*, *do not remember*. Attending a dental check-up within the last year was defined as preventive care use. Respondents who chose the options ‘*more than 1 to 2 years ago*, *more than 2 to 5 years ago*, *more than 5 years*, *never*, *do not remember*’ were classified as not having undertaken preventive dental care.

7. *Smoking habits*: The questionnaire requested information on the respondents’ habits of cigarette smoking separately. The questions had six alternatives - *No*, *never*, *No*, *I used to*, *but I quit*, *Yes*, *once a month or less*, *Yes*, *a few times* (*2*–*3*) *a month*, *Yes*, *a few times* (*2*–*3*) *a week*, *Yes*, *once a day or more*. To dichotomise the variable, those who reported no present smoking habits were considered as non-smokers. All those who chose options ‘*Yes*, *once a month or less*, *Yes*, *a few times* (*2*–*3*) *a month*, *Yes*, *a few times* (*2*–*3*) *a week*, *Yes*, *once a day or more*’ were classified as smokers.

### Statistical analysis

Chi-square test was used to test for significant differences between subgroups. Binary logistic regression models were fitted to the data to calculate odds ratios (OR) and confidence intervals (95% CI) for each of the four oral self-care measures. The independent variables for the model were sex and age. Age was dichotomised using the median age as the point of dichotomisation. The median age for the study group was 11 years. Children 8–10 years were referred to as the younger age group while those 11 to 16 were referred to as the older age group. The binary logistic regression model was used to calculate the association of the independent variables with dependent variables (tooth brushing more than once a day, intake of sugary snacks less than once a day, regular use of fluoride toothpaste, and use of dental floss every day or more). Association between the independent variables and recommended oral self-care was also assessed. STATA version 10 was used for data processing and statistical analysis.

### Ethical consideration

Approval was obtained from the Research and Ethics Committee of University of Nigeria Teaching Hospital, Enugu, and the University College Hospital, Ibadan. Permission was also sought from the State Ministry of Education via the Schools Management Board in the Local Government Area, and the Head of the various schools.

## Results

A total of 2,676 (1,223 males and 1,453 females) pupils were recruited into the study. This represents 4.5% more than the minimum sample size required for the study. None of the students who met the eligibility criteria for study participation refused to participate in the study. The mean age for the group was 11.1 ± 2.39 yrs while the median age for the group was 11 years. There were 1,211 and 1,465 pupils categorized as younger and older age group respectively.

Most respondents (2,209 - 82.5%) reported frequent use of fluoride containing toothpastes. More younger than older pupils reported the use of fluoride containing toothpaste (92.7% vs 74.1%; p < 0.001). Most of the respondents (55.7%) reported once a day brushing with only 31.5% of respondents brushing more than once a day. There was no age group (p = 0.6) nor sex (p = 0.5) differences observed for those who brushed more than once a day. Less than a quarter of the respondents (22.6%) had ever used the dental floss. More older than younger respondents use the dental floss once a day or more when compared (14.4% vs 3.5%; p < 0.001). Also more older than younger respondents (34.7% vs 24.9%; p < 0.001) and more females than males (34.4% vs 25.4%; p < 0.001) ate sugar-containing snacks less than once a day. See Table 1.

**Table 1:** Number and percentage of school children who reported preventive oral health practices (N = 1863)

**Variables: preventive oral health practices**	**Age**		**P value**	**Sex**		**P value**	
	**8–10 yrs**	**11–16 yrs**		**Male**	**Female**		**Total**
	**N = 1,211**	**N = 1,465**		**N = 1,224**	**N = 1,452**		**N = 2,676**
1 Brushes more than once a day	394 (32.5%)	450 (30.7%)	0.60	463 (37.8%)	481 (33.1%)	0.15	844 (31.5%)
2 Use fluoridated toothpaste always or almost always	1,123 (92.7%)	1,086 (74.1%)	**0.000**	1,017 (83.1%)	1,192 (82.1%)	0.50	2,209 (82.5%)
3 Eat sugar-containing snacks less than once a day	301 (24.9%)	508 (34.7%)	**0.000**	311 (25.4%)	499 (34.4%)	**0.000**	809 (30.2%)
**Practice of recommended oral self-care	90 (7.4%)	119 (8.1%)	**0.000**	84 (6.9%)	125 (8.6%)	**0.03**	209 (7.8%)
4 Floss at least once a day	42 (3.5%)	211 (14.4%)	**0.000**	113 (9.2%)	140 (9.6%)	0.72	253 (9.5%)
5 Dental check-up within last 1 year	100 (8.3%)	239 (16.3%)	**0.000**	178 (14.5%)	161 (11.1%)	**0.007**	339 (12.7%)
6 No present smoking habit	1,201 (99.2%)	1,265 (86.3%)	**0.000**	1,131 (92.4%)	1,335 (91.9%)	0.61	2,466 (92.2%)

**Variables 1, 2 and 3 constituted the index "Practice of recommended self-care".

Overall, only 7.8% of the study population practiced the recommended oral self-care: More older than younger pupils (8.1% vs 7.4%; p = <0.001) and more females than males (8.6% vs 6.9%; p = 0.03) practiced the recommended oral self-care (Table 1).

Very few respondents (12.7%) had visited the dental clinic for a check-up in the last one year preceding the survey. More older than younger respondents (16.3% vs 8.3%; p < 0.001) and more males than females (14.5% vs 11.1%; p = 0.007) had visited the clinic in the last one year. Also, majority of the respondents (92.2%) were non-smokers with more younger than older respondents (99.2% vs 86.3%; p = <.001) being non-smokers.

Tables 2 and 3 shows a summary of the predictors of preventive oral health practices by respondents. Older adolescents had an 8.0% increased odds (aOR: 1.08; CI:0.81–1.43; p = 0.61) and males had a 20.0% decreased odds (aOR: 0.80; CI:0.60-1.06; p = 0.12) of practicing recommended oral self-care though observed differences were not statistically significant. Older adolescents had significantly increased odds of using flossing once a day (aOR: 4.69; CI:3.33 – 6.59; p < 0.001), taking in between meals snacks less than once a day (aOR:1.57; CI:1.33 – 1.87; p < 0.001), vising the dentist at least once a year (aOR:2.14; CI:1.68-2.73; p < 0.001), and being a smoker (aOR:21.01; CI:10.72-41.17; p < 0.001); and a significant decreased odds of using fluoridated toothpaste always (aOR:0.22; CI:0.18-0.29; p < 0.001) when compared to younger adolescents. Also, males had a decreased odds of taking in between meals snacks less than once a day (aOR:0.66; CI:0.55-0.78; p < 0.001) and an increased odds of visiting the dentist at least once a year (aOR:1.44; CI:1.11-1.76 p = 0.004) when compared with females.

**Table 2:** Predictor for use of preventive oral health practices by age for school children in Southern Nigeria (N = 1863)

	**Age**		**Unadjusted OR (CI)**	**p-Value**	**Sex adjusted OR (CI)**	**p-Value**
	**8–10 yr (Ref. group)**	**11–16 yr**				
**Brushing**						
**Once a day or less 1832 (68.5%)**	817 (67.4%)	1015 (69.3%)				
**More than once a day 845 (31.5%)**	395 (32.6%)	450 (30.7%)	0.92 (0.78 – 1.08)	0.30	0.91 (0.77 – 1.07)	0.27
**Fluoride tooth paste**						
**Not always 467 (17.4%)**	88 (7.3%)	379 (25.9%)				
**Always 2210 (82.5%)**	1124 (92.7%)	1086 (74.1%)	0.22 (0.18 – 0.28)	**<0.001**	0.22 (0.18 – 0.29)	**<0.001**
**Snaking in between meals**						
**Once a day and more 1866 (69.6%)**	909 (75.0%)	957 (65.3%)				
**Less than once a day 811 (30.2%)**	303 (25.0%)	508 (34.7%)	1.59 (1.35 –1.89)	**<0.001**	1.57 (1.33 – 1.87)	**<0.001**
**Practice of recommended oral self**-**care**						
**Not perfect 2465 (92.2%)**	1120 (92.4%)	1345 (91.8%)				
**Perfect 212 (7.8%)**	92 (7.6%)	120 (8.2%)	1.09 (0.82 – 1.44)	0.57	1.08 (0.81 – 1.43)	0.61
**Dental visit (Check-up)**						
**Less than once a year 2331 (87.3%)**	1108 (91.4%)	1223 (83.5%)				
**At least once a year 346 (12.7%)**	104 (8.6%)	242 (16.5%)	2.11 (1.65 – 2.69)	**<0.001**	2.14 (1.68 – 2.73)	**<0.001**
**Flossing**						
**Less than once a day 2424 (90.5%)**	1170 (96.5%)	1254 (85.6%)				
**At least once a day 253 (9.5%)**	42 (3.5%)	211 (14.4%)	4.69 (3.33 – 6.59)	**<0.001**	4.69 (3.33 – 6.59)	**<0.001**
**Smoking**						
**Not Current smoker 2469 (92.2%)**	1203 (99.3%)	1266 (86.4%)				
**Smoker 208 (7.8%)**	9 (0.7%)	199 (13.6%)	21.01 (10.72 – 41.16	**<0.001**	21.01 (10.72 – 41.17)	**<0.001**

**Table 3:** Predictor for use of preventive oral health practices by sex for school children in Southern Nigeria (N = 1863)

	**Sex**		**Unadjusted OR (CI)**	**p-Value**	Age* adjusted OR (CI)	**p-Value**
	**Female (Ref. group)**	**Male**				
**Brushing**						
**Once a day or less 1832 (68.5%)**	972 (66.9%)	860 (70.3%)				
**More than once a day 845 (31.5%)**	481 (33.1%)	364 (29.7%)	0.86 (0.73 – 1.01)	0.06	0.85 (0.72 – 1.00)	0.06
**Flouride tooth paste**						
**Not always 467 (17.4%)**	260 (17.9%)	207 (16.9%)				
**Always 2210 (82.5%)**	1193 (82.1%)	1017 (83.1%)	1.07 (0.88 – 1.31)	0.51	1.02 (0.83 – 1.26)	0.84
**Flossing**						
**Less than once a day 2424 (90.5%)**	1313 (90.4%)	1111 (90.8%)				
**At least once a day 253 (9.5%)**	140 (9.6%)	113 (9.2%)	0.95 (0.74 – 1.24)	0.72	1.00 (0.77 – 1.30)	1.00
**Practice of recommended oral self**-**care**						
**Not perfect 2465 (92.2%)**	1327 (91.3%)	1138 (93.0%)				
**Perfect 212 (7.8%)**	126 (8.7%)	86 (7.0%)	0.80 (0.60 – 1.06)	0.12	0.80 (0.60 – 1.06)	0.12
**Snaking in**-**between meals**						
**Once a day and more 1866 (69.8%)**	953 (65.6%)	913 (74.6%)				
**Less than once a day 811 (30.2%)**	500 (34.4%)	311 (25.4%)	0.65 (0.55 – 0.77)	**<0.001**	0.66 (0.55 – 0.78)	**<0.001**
**Dental visit (Check-up)**						
**Less than once a year 2331 (87.3%)**	1288 (88.6%)	1043 (85.2%)				
**At least once a year 346 (12.7%)**	165 (11.4%)	181 (14.8%)	1.35 (1.08 – 1.70)	**0.009**	1.40 (1.11 – 1.76)	**0.004**
**Smoking**						
**Not Current smoker 2469 (92.2%)**	1337 (92.0%)	1132 (92.5%)				
**Smoker 208 (7.8%)**	116 (8.0%)	92 (7.5%)	0.94 (0.70 – 1.25)	0.65	1.00 (0.75 – 1.34)	0.99

*Age as a dichotomous variable (8–10 years vs. 11–16 years).

## Discussion

This study was able to highlight that the use of fluoride containing toothpaste was widespread among the study population. However, the practice of twice daily tooth brushing and the use of dental floss were low. Also, less than a third of the respondents consumed sugar less than once a day. These findings may most likely be a true reflection of the status of oral health practices in the population based on the strength of the methodology used in this study. First, the responses were anonymous which enhanced confidentiality. The issue of confidentiality was also highlighted to the respondents when the study was introduced by the researchers in the field. Secondly, the students filled the questionnaire themselves therefore reducing the changes of response bias that may have resulted with an interviewer administered questionnaire.

This study highlights some interesting findings. Combination of approaches is important for successful behaviour dependent interventions [31] as is the case with caries prevention [32]. The most important caries preventive oral health practice for children is a combination of assured continuous use of fluoridated toothpaste once a day or more [16], and restricted intake of refined carbohydrate. Unfortunately, an extremely low number of study respondents use a combination of these caries prevention approaches.

The literature discusses extensively about the role of individual factors for the prevention and control of caries in children. Unfortunately, very little is known about the efficacy and practices of a combination of these approaches in children. There have been prior studies on combination approach for caries prevention in dental students in Iran [32], Nigeria [33], and Mongolia [34], and among dental educators in Mongolia [17]. The authors could find no literature discussing the use of a combination of preventive oral health care practices in children. Although age and sex were not significant predictors of the use of recommended oral self-care, they were significant predictors of use of some preventive oral health care practices as enumerated in Tables 2 and 3. Older adolescents seem to have better preventive oral health care practices (flossing, dental visits, snacking) than younger adolescents.

Regular utilization of oral health centres is also a hallmark for preventive oral health care. Consistently, the literatures show poor dental service utilization by children in Nigeria [35,36], and high rates of untreated caries with its attendant problems [23,24]. Regular dental service utilization was low in this study indicating a need to identify and institute actions that could facilitate access of pupils to dental care. Males had had more annual dental visits than females. The reasons for this is not quite clear: visits may have resulted from the need for curative treatment since they have greater risk for caries resulting from worse snacking habits when compared to females. A prior study had shown that females had deceased caries risk when compared to males [37].

Tobacco smoking is an important public health issue having been implicated as a major risk factor for a range of oral diseases like periodontal disease, leukoplakia, leukoedema and oral cancer. Many smokers start before the age of 18 years [38]. This study showed that a larger proportion of older than younger pupils smoke. The direction of this observation cannot be readily deciphered from the results of this study (whether smoking increases with age or whether smoking tendencies have decreased with time). It is however important to understand the direction of this observation so as to identify necessary interventions if the association observed is an increase in tendency to smoke with increasing age.

Understanding the implication of the findings from this study may indeed inform design of public oral health programmes targeting school pupils in Southern Nigeria. The data seem to suggest that preventive oral health habits are picked up in later years which may be through other forms of socialization outside the home. These teenagers may have learnt about appropriate oral health care practices through the secondary socialization process. It will be important to understand how pupils learn about oral health care, and then effectively use of these media to reach out to children and adolescents. The reasons for and implications of the sex differences observed with use of recommended oral self-care in this study will need to be explored further using possibly qualitative study methods. Prior studies in Nigeria had shown that oral hygiene and oral hygiene practices are better in females than males [39,40]. The explanation for this observation remains unknown.

This study has its limitations. First is the inability to generalize the outcome of this study to all children in Southern Nigerian as a significant number of children who are not in school and those not resident in urban areas were not included the study sample. Secondly, data was not captured on the possible role of the socio-economic status of the child – a factor that would influence the ability to purchase many of the commodities needed for preventive oral self-care - as a potential limiting factor to the use of recommended self-care. Third, the sample size for the study was generated using the prevalence of caries - an outcome variable for the study – rather than the prevalence of preventive self-care care practice thereby introducing a type 1 error into the study. Future studies on oral health issues in children in Nigeria would better be conducted using a household survey to ensure enrolment of both in and out of school children and adolescents.

## Conclusions

In conclusion, the use of recommended oral self-care measures for caries prevention in this study population was low due to low number of persons who brush twice daily and the high number of persons who consume sugar-containing snacks or drinks between main meals daily or more. School based programmes can therefore focus on addressing these factors. Age and sex were predictive factors for the use of components of the oral self-care measures. Future studies would be required to understand ‘why’ and ‘how’ age and sex impacts on the use of caries preventive oral measures so as to be able to design effective prevention programmes for the study population.

## Competing interests

The authors declare that they have no competing interest.

## Authors’ contributions

MOF: initiated the study, made substantial contributions to conception, design, acquisition, analysis and interpretation of data for this study; has been involved in drafting and revising the manuscript for important intellectual content; and has given final approval of the version to be published. MRK: made substantial contributions to design, and interpretation of data for this study; has been involved in drafting and revising the manuscript for important intellectual content; and has given final approval of the version to be published. BOP: made contributions to design, acquisition, analysis and interpretation of data for this study; has been involved in drafting and revising the manuscript for important intellectual content; and has given final approval of the version to be published. NO: made substantial contributions to design, acquisition, analysis and interpretation of data for this study; has been involved in drafting and revising the manuscript for important intellectual content; and has given final approval of the version to be published. YIA: made contributions to acquisition of data for this study; has been involved in drafting and revising the manuscript for important intellectual content; and has given final approval of the version to be published.

## Pre-publication history

The pre-publication history for this paper can be accessed here:

http://www.biomedcentral.com/1472-6831/14/83/prepub

## References

[B1] Petersen PE The World Oral Health Report 2003: continuous improvement of oral health in the 21st century–the approach of the WHO Global Oral Health Programme Community Dent Oral Epidemiol 2003 31 Suppl 1 3 23 15015736 10.1046/j..2003.com122.x

[B2] Inglehart M Tedesco LA Behavioural research related to oral hygiene practices: new century model of oral health promotion Periodontol 2000 1995 8 15 23 9567943 10.1111/j.1600-0757.1995.tb00042.x

[B3] Schou L The relevance of behavioural sciences in dental practice Int Dent J 2000 50 324 332 11197193 10.1111/j.1875-595x.2000.tb00582.x

[B4] Axelsson P Albandar JM Rams TE Prevention and control of periodontal diseases in developing and industrialized nations Periodontol 2000 2002 29 235 246 12102711 10.1034/j.1600-0757.2002.290112.x

[B5] Löe H Oral hygiene in the prevention of caries and periodontal disease Int Dent J 2000 50 129 139 10967765 10.1111/j.1875-595x.2000.tb00553.x

[B6] Richards W Ameen J The impact of attendance patterns on oral health in a general dental practice Br Dent J 2002 193 697 702 12529726 10.1038/sj.bdj.4801664

[B7] Ismail AI Lewis DW Dingle JL Prevention of periodontal disease Canadian Task Force on the Periodic Health Examination. Canadian Guide to Clinical Preventive Health Care 1994 Ottawa: Health Canada 420 431

[B8] Nyvad B Fejerskov O, Kidd E The role of oral hygiene Dental caries, the disease and its clinical management 2003 Oxford: Blackwell Munksgaard 171 176

[B9] Marinho VC Higgins JP Sheiham A Logan S Fluoride toothpastes for preventing dental caries in children and adolescents Cochrane Database Syst Rev 2003 1 CD002278 12535435 10.1002/14651858.CD002278 PMC8439270

[B10] Twetman S Axelsson S Dahlgren H Holm AK Källestål C Lagerlöf F Lingström P Mejàre I Nordenram G Norlund A Petersson LG Söder B Caries-preventive effect of fluoride toothpaste: a systematic review Acta Odontol Scand 2003 61 347 355 14960006 10.1080/00016350310007590

[B11] Bratthall D Hansel-Petersson G Sundberg H Reasons for the caries decline: what do the experts believe? Eur J Oral Sci 1996 104 416 422 8930592 10.1111/j.1600-0722.1996.tb00104.x

[B12] Burt BA Prevention policies in the light of the changed distribution of dental caries Acta Odontol Scand 1998 56 179 186 9688230 10.1080/000163598422956

[B13] Adair SM Evidence-based use of fluoride in contemporary pediatric dental practice Pediatr Dent 2006 28 133 142 16708788

[B14] Davies RM The prevention of dental caries and periodontal disease from the cradle to the grave: what is the best available evidence? Dent Update 2003 30 170 176 12830693 10.12968/denu.2003.30.4.170

[B15] Burt BA Pai S Sugar consumption and caries risk: a systematic review J Dent Educ 2001 65 1017 1023 11699972

[B16] Kidd E Fejerskov O Changing concepts in cariology: forty years on Dent Update 2013 40 277 278 280–82, 285–286 23829008 10.12968/denu.2013.40.4.277

[B17] Tseveenjav B Vehkalahti M Murtomaa H Oral health and its determinants among Mongolian dentists Acta Odontol Scand 2004 62 1 6 15124776 10.1080/00016350310008003

[B18] Reibel J Tobacco and oral diseases. Update on the evidence, with recommendations Med Princ Pract 2003 12 22 32 12707498 10.1159/000069845

[B19] Petersen PE Tobacco and oral health–the role of the world health organization Oral Health Prev Dent 2003 1 309 315 15643759

[B20] Squier CA Smokeless tobacco and oral cancer: a cause for concern? CA Cancer J Clin 1984 34 242 247 6432237 10.3322/canjclin.34.5.242

[B21] Mashberg A Boffetta P Winkelman R Garfinkel L Tobacco smoking, alcohol drinking, and cancer of the oral cavity and oropharynx among U.S. veterans Cancer 1993 72 1369 1375 8339227 10.1002/1097-0142(19930815)72:4<1369::aid-cncr2820720436>3.0.co;2-l

[B22] Folayan MO Khami MR Folaranmi N Orenuga O Popoola BO Olatosi O Ligali TO Sofola OO Adeniyi AA Esan A Osaguona A Determinants of preventive dental practice for children among final year dental students in Nigeria Int J Paediatr Dent 2013 doi:10.1111/ipd.12024 10.1111/ipd.12024 23414142

[B23] Oziegbe EO Esan TA Prevalence and clinical consequences of untreated dental caries using PUFA index in suburban Nigerian school children Eur Arch Paediatr Dent 2013 14 4 227 231 23780656 10.1007/s40368-013-0052-5

[B24] Folayan MO Sofola OO Oginni AB Caries incidence in a cohort of primary school students in Lagos State, Nigeria followed up over a 3 years period Eur Arch Paediatr Dent 2012 13 312 318 23235132 10.1007/BF03320833

[B25] World Health Organization Global action plan for the prevention and control of non-communicable diseases 2013–2020 2013 Available at: http://www.who.int/nmh/events/2013/revised_draft_ncd_action_plan.pdf. Accessed 6th March, 2014

[B26] Nigeria Education Fact Sheet 2013 Available at: http://photos.state.gov/libraries/nigeria/487468/pdfs/JanuaryEducationFactSheet.pdf Accessed 15th May 2013

[B27] Nigeria: School Enrolment 2013 Available at: http://www.indexmundi.com/facts/nigeria/school-enrollment. Accessed 15th May 2013

[B28] Akpata KS Oral health in Nigeria Int Dent J 2004 54 361 366 15631097 10.1111/j.1875-595x.2004.tb00012.x

[B29] Okoye LO Chukweneke FN Akaji EA Folaranmi N Caries experience among school children in Enugu, Nigeria J Coll Med 2010 14 17 23

[B30] Denloye OO Ajayi D Bankole O A study of dental caries prevalence in 12–14 year old school children in Ibadan, Nigeria Paediatr Dent J 2005 15 147 151

[B31] Jafa K A winning approach: combination interventions for greater impact Impact Magazine Issue 10 Available at: http://psiimpact.com/a-winning-approach-combining-interventions-for-greater-impact/. Accessed 4th of April, 2014

[B32] Khami MR Virtanen JI Jafarian M Murtomaa H Oral health behaviour and its determinants amongst Iranian dental students Eur J Dent Educ 2007 11 42 47 17227395 10.1111/j.1600-0579.2007.00424.x

[B33] Folayan MO Khami MR Folaranmi N Popoola BO Sofola OO Ligali TO Esan AO Orenuga OO Determinants of preventive oral health behaviour among senior dental students in Nigeria BMC Oral Health 2013 13 28 23777298 10.1186/1472-6831-13-28 PMC3700852

[B34] Tseveenjav B Vehkalahti M Murtomaa H Preventive practice of Mongolian dental students Eur J Dent Educ 2002 6 74 78 11975669 10.1034/j.1600-0579.2002.60206.x

[B35] Ola DO Gambôa ABO Folayan MO Marcene W Family structure, socio-economic position and oral health services utilisation in Nigerian senior secondary school pupils J Public Health Dent 2012 doi:10.1111/j.1752-7325.2012.00362.x 10.1111/j.1752-7325.2012.00362.x 22970821

[B36] Adekoya-Sofowora CA The effect of poverty on access to oral health care in Nigeria Niger Dent J 2008 16 40 42

[B37] Folayan MO Sowole A Kola-Jebutu A Risk factors for caries in Nigerian children J Clin Paediatr Dent 2008 32 171 175 10.17796/jcpd.32.2.p1rq2x4u5744800g 18389686

[B38] Secretary to State for Health and Secretaries of State for Scotland, Wales and Northern Ireland Smoking Kills A White Paper on Tobacco London 1999 HM Stationery Office

[B39] Kolawole KA Oziegbe EO Bamise CT Oral hygiene measures and the periodontal status of school children Int J Dent Hyg 2011 9 143 148 doi:10.1111/j.1601-5037.2010.00466.x. Epub 2011 Marh 1 21356014 10.1111/j.1601-5037.2010.00466.x

[B40] Agbelusi GA Jeboda SO Oral health status of 12-year-old Nigerian children West Afr J Med 2006 25 195 198 17191418 10.4314/wajm.v25i3.28277

